# Supplementary figures and images for: Identification of hidden N4-like viruses and their interactions with hosts
Source: mSystems. 2023 Sep 13;8(5):e00197-23. doi: 10.1128/msystems.00197-23 (PMC10654107; doi:10.1128/msystems.00197-23)

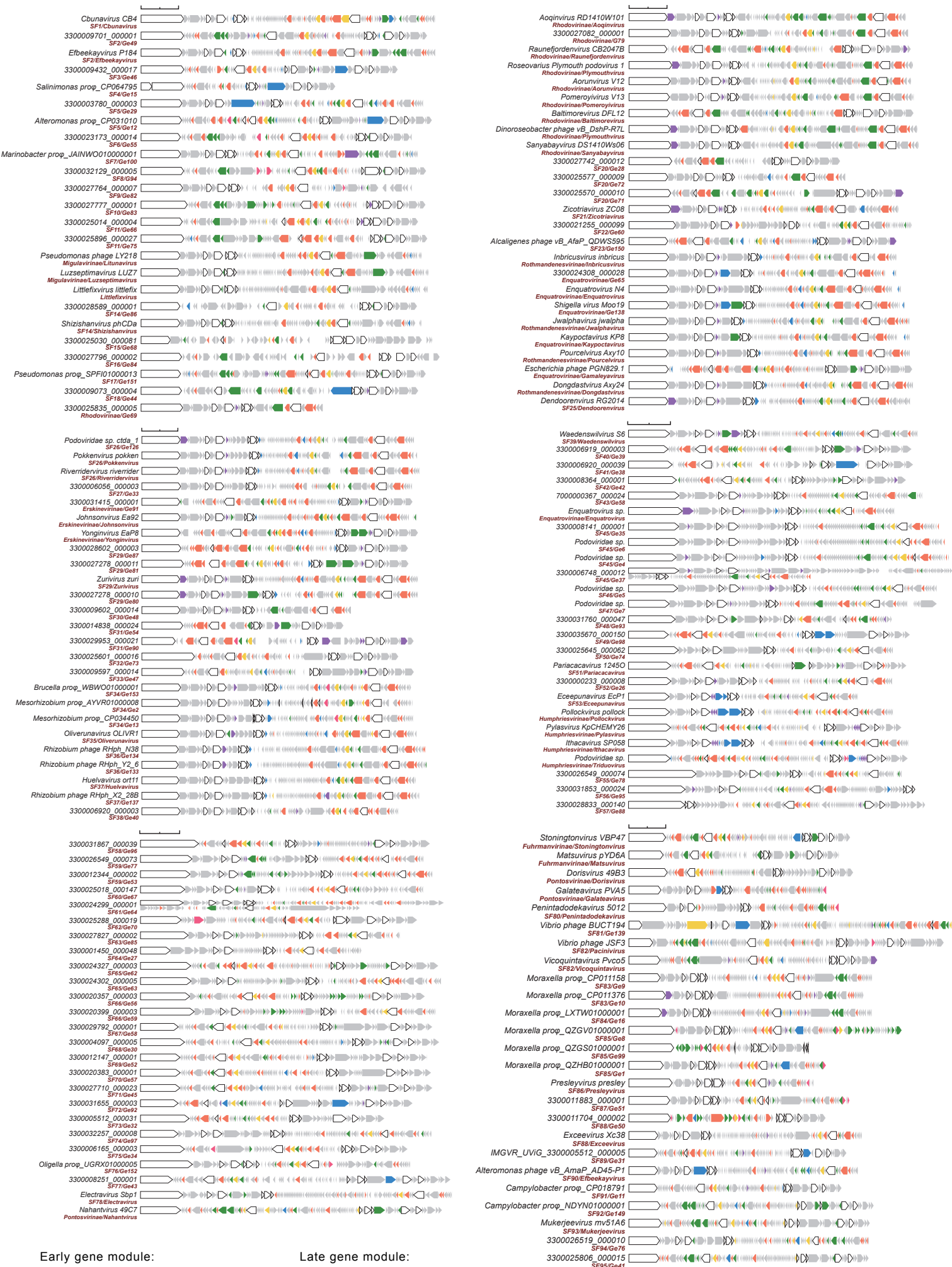

Supplement: Fig. S1 — The genome organization of N4-like viruses. [file msystems.00197-23-s0001.pdf]

A

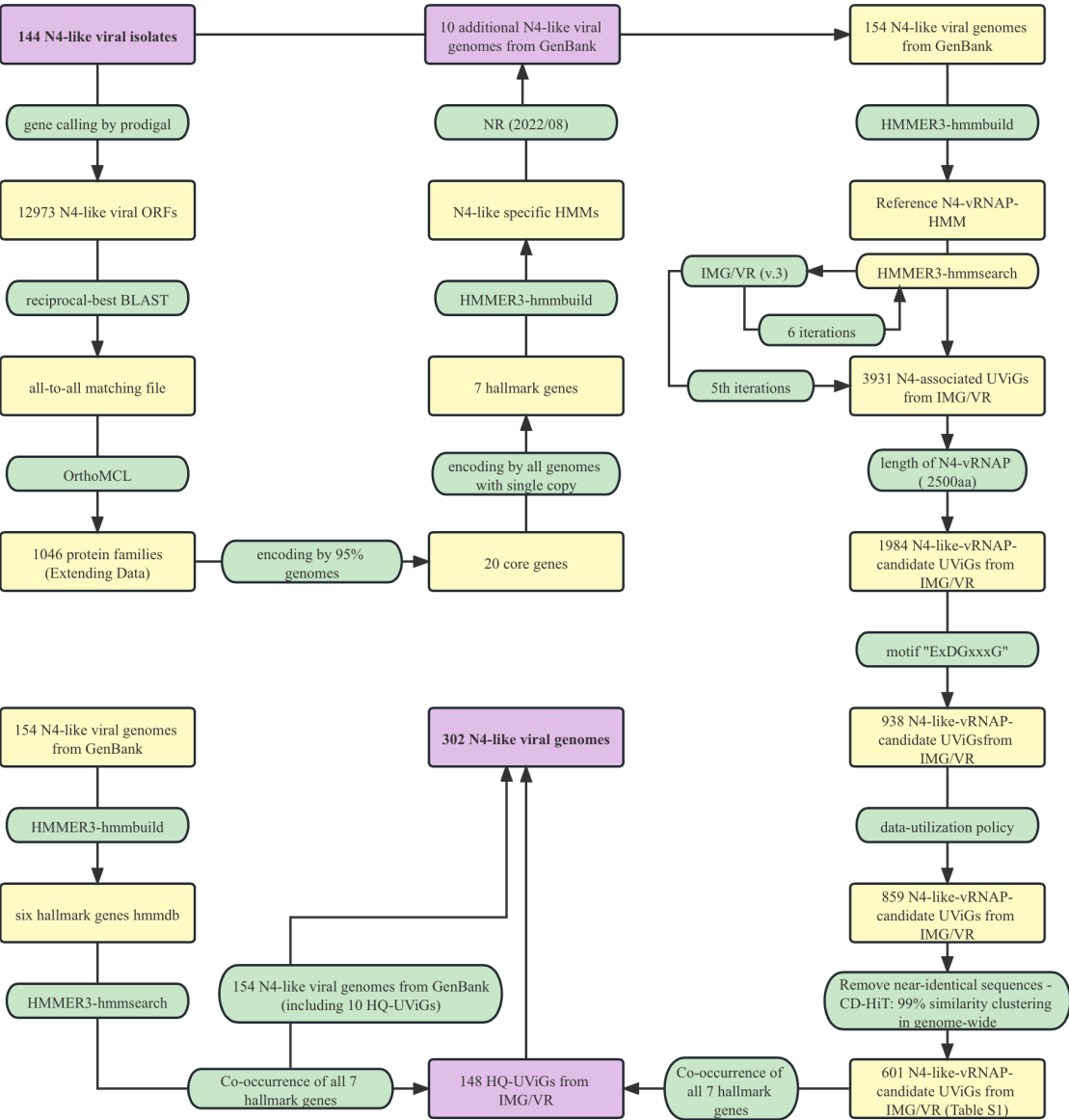

B

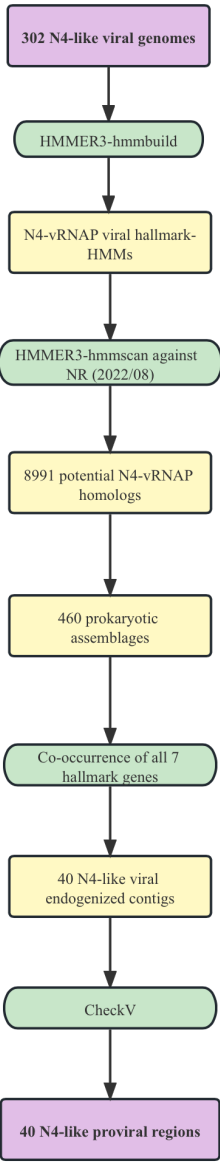

Supplement: Fig. S2 — Identification pipeline for N4-like viruses. [file msystems.00197-23-s0002.pdf]
