# Supplementary material for: Identification of hidden N4-like viruses and their interactions with hosts
Source: mSystems. 2023 Sep 13;8(5):e00197-23. doi: 10.1128/msystems.00197-23 (PMC10654107; doi:10.1128/msystems.00197-23)
Supplement: Supplemental text — Legends for supplemental figures. [file msystems.00197-23-s0003.docx]

**Supplementary materials**

To mSystemes:

**Identification of hidden N4-like viruses and their interactions with hosts**

Kaiyang Zheng^1,2*^; Yantao Liang^1,2*^†; David Paez-Espino^3,4*^; Xiao Zou^5^; Chen Gao^1,2^; Hongbing Shao^1,2^; Yeong Yik Sung^2,6^; Wen Jye Mok^2,6^; Li Lian Wong^2,6^; Yu-Zhong Zhang^1,7^; Jiwei Tian^8^; Feng Chen^9^; Nianzhi Jiao^10^; Curtis A. Suttle^11^; Jianfeng He^12^†; Andrew McMinn^1,13^†; Min Wang^1,2,14^†

1. College of Marine Life Sciences; Institute of Evolution and Marine Biodiversity; Frontiers Science Center for Deep Ocean Multispheres and Earth System; Key Lab of Polar Oceanography and Global Ocean Change, Ocean University of China, Qingdao 266003, China.
2. UMT-OUC Joint Centre for Marine Studies, Qingdao 266003, China.
3. DOE Joint Genome Institute, Lawrence Berkeley National Laboratory, Berkeley, CA, USA
4. Mammoth Biosciences, Inc., South San Francisco, CA, USA
5. Qingdao Central Hospital, Qingdao 266042, China
6. Institute of Marine Biotechnology, Universiti Malaysia Terengganu (UMT), 21030, Kuala Nerus, Malaysia.
7. State Key Laboratory of Microbial Technology; Marine Biotechnology Research Center, Shandong University, Qingdao 266237, China
8. Key Laboratory of Physical Oceanography; Ministry of Education; Ocean University of China, Qingdao 266100, China
9. Institute of Marine and Environmental Technology, University of Maryland Center for Environmental Science, Baltimore, Maryland, USA
10. Institute of Marine Microbes and Ecospheres; State Key Laboratory of Marine Environmental Sciences, Xiamen University, 361005, China
11. Departments of Earth; Ocean and Atmospheric Sciences; Microbiology and Immunology; Botany and Institute for the Oceans and Fisheries, The University of British Columbia, Vancouver, British Columbia BC V6T 1Z4, Canada
12. SOA Key Laboratory for Polar Science, Polar Research Institute of China, Shanghai

200136, China

1. Institute for Marine and Antarctic Studies, University of Tasmania, Hobart, Tasmania 7001, Australia
2. The Affiliated Hospital of Qingdao University, Qingdao 266003, China

*These authors contributed equally to this work.

†Corresponding authors. Email: liangyantao@ouc.edu.cn (Y.L.); [hejianfeng@pric.org.cn](mailto:hejianfeng@pric.org.cn) (J.H.); [andrew.mcminn@utas.edu.au](mailto:andrew.mcminn@utas.edu.au) (1); mingwang@ouc.edu.cn (M.W.)

# Supplementary figures

Fig. S1 The genome organization of N4-like viruses. One N4-like viral genomes with largest genome size from per genus/proposed genus was selected as the representative genome diagram showing here. Open reading frames (ORFs) of these genomes were assigned into six classifications, which are indicated by different colors. The seven ORFs of N4-like viral hallmark genes are highlighted in bold lines. The genomic scales are displayed at the top of each sub-module.

Fig. S2 Identification pipeline for N4-like viruses. The workflow includes two major parts: detection of N4-like uncultured viral genomes (UViGs) (A), and integrated high-quality proviral regions (B). This led to 795 N4-like UViGs (encoding virion-encapsulated RNA polymerase). Boxes in yellow and green represent generated results and corresponding processing steps. Boxes in purple represent the final results that were used for further analysis in this study. The important datapoints, including number of used initial reference genomes, number of generated HQ N4-like viral genomes, number of HQ N4-like proviruses, are highlighted in bold font.
